# Drug-drug interaction perpetrators of oxycodone in patients with cancer: frequency and clinical relevance

**DOI:** 10.1007/s00228-023-03612-2

**Published:** 2024-01-13

**Authors:** L. M. G. Hulskotte, W. Töpfer, A. K. L. Reyners, K. Taxis, F. G. A. Jansman

**Affiliations:** 1 Department of Clinical Pharmacy, Deventer Teaching Hospital, Nico, Bolkesteinlaan 75, 7416 SE Deventer, the Netherlands; 2 Unit of PharmacoTherapy, -Epidemiology &-Economics, Groningen Research Institute of Pharmacy (GRIP), University of Groningen, Groningen, the Netherlands; 3 Department of Medical Oncology, University of Groningen, University Medical Center Groningen, Groningen, the Netherlands

**Keywords:** CYP3A4, CYP2D6, Drug-drug interactions, Oncology, Oxycodone, Pharmacokinetics

## Abstract

**Aim:**

Oxycodone is known to have numerous drug-drug interactions (DDIs) that can potentially decrease efficacy or lead to adverse drug reactions (ADRs). However, there is limited research on the frequency of DDIs associated with oxycodone, which is important in optimising pharmacovigilance and the need for additional research on certain DDIs. In this study, the frequency of pharmacologically and clinically relevant DDI perpetrators was studied in patients with cancer.

**Methods:**

This was a cross-sectional study using hospital pharmacy records of patients with cancer who were prescribed oxycodone between September 2021 and September 2022. Medication records of patients prescribed oxycodone during a period of ≥ 5 consecutive days (= oxycodone treatment episodes) were reviewed to identify the concomitant use of pharmacologically relevant perpetrators, based on reference sources (Lexicomp®, Micromedex®, the Dutch Kennisbank and the Dutch Commentaren Medicatiebewaking). The clinical relevance was examined by a clinical pharmacologist and a medical oncologist. Additionally, the frequency of double interactions—concomitant oxycodone use with two CYP3A4 and / or CYP2D6 perpetrators—was studied.

**Results:**

Overall, 254 oxycodone treatment episodes were included, of which 227 (89.4%) were found to contain at least one pharmacologically relevant DDI perpetrator. Of these, 210 (82.7%) were considered to be clinically relevant. A total of 80 different pharmacologically relevant perpetrators were identified, with 65 (81.3%) being considered clinically relevant. Double interactions were observed in 21 (8.3%) oxycodone treatment episodes.

**Conclusion:**

A high frequency of pharmacologically and clinically relevant perpetrators of oxycodone was observed in our cohort. Moreover, a high number of double interactions involving oxycodone was registered. More intense monitoring of DDIs may be needed to improve medication safety of patients with cancer taking oxycodone.

**Supplementary Information:**

The online version contains supplementary material available at 10.1007/s00228-023-03612-2.

## Introduction

Pain is a common symptom among patients with cancer. The World Health Organization (WHO) reports that 66% of patients with advanced metastatic or terminal cancer experience pain [1]. Adequate pain management is crucial in order to maintain a good quality of life [2, 3]. The WHO has established guidelines for adequate relief of cancer-related pain, including the use of opioids to treat moderate-to-severe cancer-related and neuropathic pain [1, 4]. Oxycodone, the second most-consumed opioid worldwide, is also commonly prescribed in the Netherlands [5, 6].

Oxycodone is a semisynthetic µ-receptor agonist that is primarily metabolised by CYP3A4 into noroxycodone and by CYP2D6 into oxymorphone [7, 8]. Due to its metabolism via cytochrome P450 (CYP) enzymes, oxycodone is susceptible to pharmacokinetic drug interactions [9]. For example, CYP3A4 inhibition increases the plasma concentration of oxycodone significantly, while CYP3A4 inducers decrease the exposure, potentially affecting its clinical efficacy [9–12]. Although the role of CYP3A4 in drug-drug interactions (DDIs) is well-established, the impact of CYP2D6-mediated drug interactions on oxycodone efficacy remains controversial [13]. Inhibition of CYP2D6 alone does not significantly increase systemic oxycodone concentrations. However, concomitant use of a CYP2D6 inhibitor (such as paroxetine) and a CYP3A4 inhibitor (such as itraconazole) greatly increases oxycodone exposure [10, 12–14]. This phenomenon, where oxycodone is concomitantly used with two CYP–enzyme-modifying perpetrators, either two CYP3A4 inducers or inhibitors, two CYP2D6 inhibitors, or a combination of CYP2D6 and CYP3A4 inhibitors, is defined as a double interaction.

Oxycodone is also susceptible to DDIs with many central nervous system (CNS) depressants. The synergistic effect of oxycodone with these drugs increases the risk of respiratory depression and oversedation [15–17]. Despite the frequent use of oxycodone and the potential for severe adverse drug reactions (ADRs) associated with DDIs involving oxycodone, there is a lack of research on the frequency of such interactions in patients with cancer.

In order to give insight in the clinical occurrence of DDIs with oxycodone and create awareness of the possible risks of DDIs with this widely used analgesic agent, the frequency of prescribing pharmacologically and clinically relevant perpetrators of oxycodone was determined in patients with cancer taking oxycodone. Additionally, the frequency of double interactions involving oxycodone was determined.

## Methods

A retrospective cross-sectional study of pharmacy records was conducted in one teaching hospital in the Netherlands. The study population consisted of patients with cancer admitted to the hospital between 1 September 2021 and 1 September 2022, identified by oncological and haematological Diagnosis Treatment Combinations (DTCs). Eligibility criteria included the presence of an oxycodone treatment episode, defined as an oxycodone prescription for a period of ≥ 5 consecutive days. Patients could have multiple oxycodone treatment episodes if they had multiple distinct episodes of oxycodone prescriptions of ≥ 5 consecutive days in the study period. This study was approved by the Medical Research Ethics Committee of Isala Clinics Zwolle, the Netherlands.

All, co-medications used during the oxycodone treatment episodes were extracted from patients’ pharmacy records. Pharmacy records from both hospital and public sector were reviewed for each patient. Drugs were included if they were prescribed for ≥ 2 consecutive days for regular or on demand use. Drugs which were only used incidentally were excluded, as for these drugs, the potential for clinically relevant interactions is low. In the case of a fixed-dose combination, each active pharmaceutical ingredient (API) was counted separately. Locally acting drugs were excluded due to their lack of interaction potential with oxycodone. Perioperative drugs were also excluded, since patients undergoing surgery are continuously monitored by healthcare professionals, rendering potential DDIs with oxycodone largely irrelevant. Data from the combined oxycodone treatment episodes has been analysed.

Data on patient demographics, including age, sex, body weight, height, and body mass index (BMI) were retrieved from pharmacy records. Additionally, information on oxycodone treatment episode characteristics, including treatment duration, maximum daily dose and the type and number of concomitantly used drugs, were collected. The maximum daily dose of oxycodone was the highest cumulative daily dose identified in a treatment episode. This was determined by adding up all individual doses of oxycodone prescribed daily. All data were extracted manually from the pharmacy records and entered into a database. This process was verified by a second investigator for accuracy.

Perpetrators of oxycodone and drugs that affect CYP3A4 and CYP2D6 enzymes were identified by consulting the following standard reference sources: two international drug interaction databases (Lexicomp® and Micromedex®) and two Dutch drug databases, i.e. the Kennisbank (managed by the Royal Dutch Society for Advancement of Pharmacy) and Commentaren Medicatiebewaking (managed by Health Base Foundation). The first two databases were selected due to their comprehensive scope, completeness, and ease of use [18]. The last two are commonly used in Dutch pharmacies, and their information is integrated in all national computerised medication surveillance systems, which generate alerts for drug interactions during the prescribing process. All drugs listed in these databases were considered pharmacologically relevant perpetrators of oxycodone, except for those with a ‘minor’ risk rating. Subsequently, the clinical relevance of the observed pharmacologically relevant perpetrators of oxycodone was assessed by the opinion of two experts—a clinical pharmacologist and a medical oncologist—as the drug interaction databases identify and rate interactions based on pharmacological mechanisms. For this purpose, a systemic and transparent risk analysis was used [19].

The number of perpetrators was registered for each individual oxycodone treatment episode. Additionally, the number of patients exposed to potential DDIs involving oxycodone was recorded. Single-dose drugs were counted separately from drugs intended for long-term use (Table S2). Lastly, double interactions were observed. The clinical relevance of double interactions was not assessed.

The data were processed using Rstudio version 2022.12.0 + 353. Patient and oxycodone treatment episode characteristics and the frequency of all outcomes were determined using descriptive statistics. Patient characteristics were tested for normality with the Shapiro–Wilk test (*p* < 0.05).

## Results

**Fig. 1:**
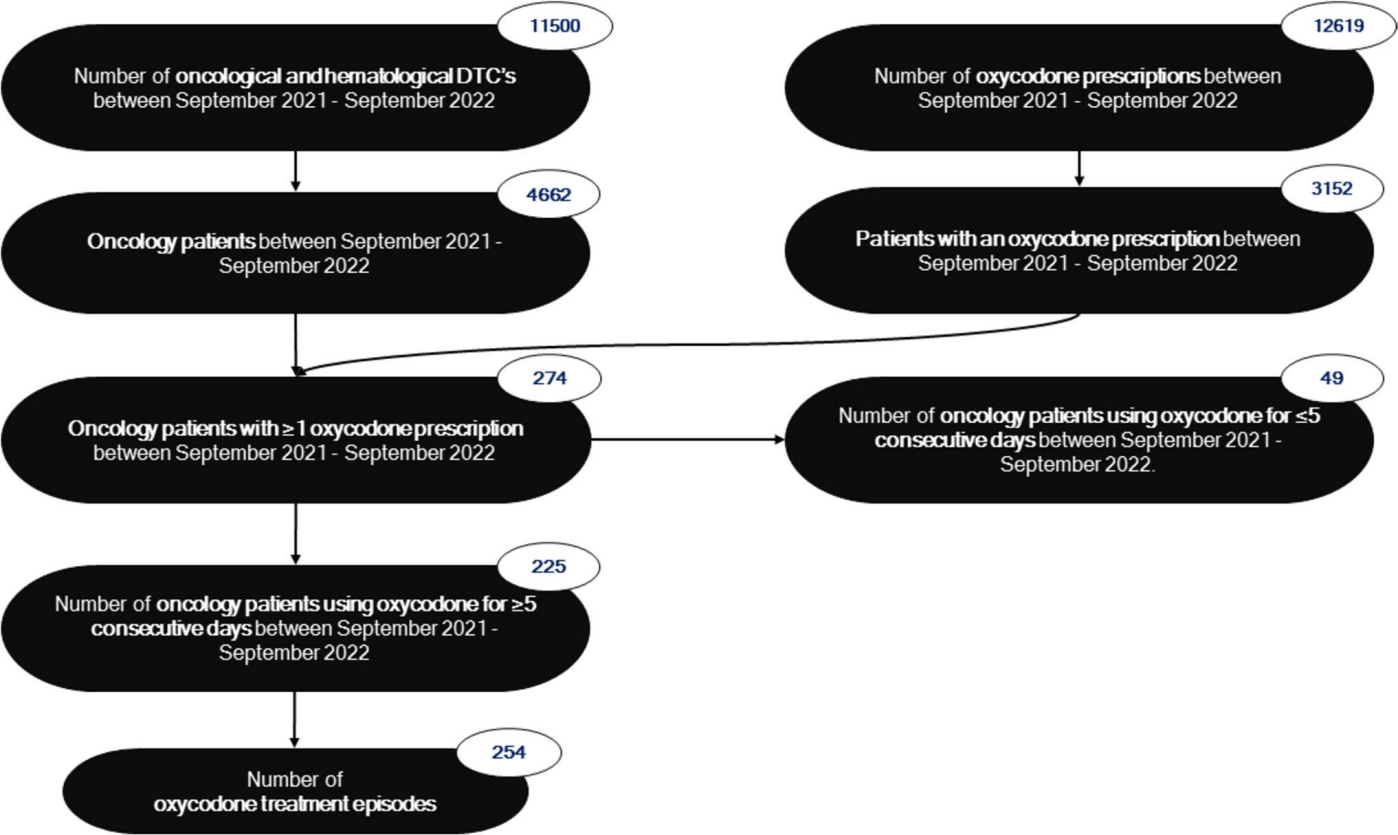
Flowchart of the in- and exclusion process. A single patient can account for multiple distinct oxycodone treatment episodes. DTC, diagnosis treatment combination

Between September 2021 and September 2022, 225 patients with cancer to whom oxycodone was prescribed for ≥ 5 consecutive days were identified. This resulted in 254 different treatment episodes, as illustrated in Fig. 1. The baseline characteristics of patients and oxycodone treatment episodes are presented in Table 1.

**Table 1:** The baseline characteristics of the patients and treatment episodes

**Characteristics**	
**Age at admission (years)**	71 [61-76]
**Sex**	-
Male (%)	126 (56)
Female (%)	99 (44)
**Body weight at admission (kg)**	80 [71-92]
**Length at admission (cm)**	173 (± 9.80)
**BMI at admission**	26.9 [23.7-30.5]
**Oxycodone treatment episode duration (days)**	46 [11-151]
**Oxycodone maximum daily dose (mg/day)**	30 [25-50]
**Number of co-medications (long-term)**	13 [9-18]

Data are presented as mean (± standard deviation) for continuous normally distributed data, median (interquartile range) for skewed continuous variables and *n* (%) for categorical data.

*BMI* body mass index

In the 254 oxycodone treatment episodes analysed, a total of 4030 drugs were used as co-medication during all of these episodes combined, consisting of 457 unique drugs. No co-medication was observed in one (0.4%) of the 254 oxycodone treatment episodes. The median number of co-medications per treatment episode was 13 (Figure S1). Of the 4030 drugs prescribed, 830 pharmacologically relevant perpetrators were identified, with 582 (70.1%) of these perpetrators also being considered clinically relevant. In 227 of the 254 oxycodone treatment episodes (89.4%), at least one pharmacologically relevant perpetrator was found, of which 210 treatment episodes contained a clinically relevant perpetrator (92.5%). The median number of pharmacologically relevant perpetrators was 3 (Figure S2). The median number of clinically relevant perpetrators was 2 (Figure S3).

Among the 80 identified perpetrators, the most common therapeutic drug classes based on anatomical therapeutic chemical (ATC) code were analgesics (such as morphine, fentanyl and piritramide; 14%), psycholeptics (such as oxazepam, temazepam, and diazepam; 12%), antidepressants (such as amitriptyline, nortriptyline, and citalopram; 9%), and antihistamines (such as clemastine, levocetirizine and cetirizine; 8%) (Table 2). The frequency of each individual perpetrator is presented in the supplementary information.

**Table 2:** The two most frequently observed drug-drug interaction perpetrators of oxycodone per ATC-class (*n* = 80). Classes containing < 2 drugs are categorised as ‘Others’ (*n* = 8). Netupitant is not included in the ATC/DDD Index and is therefore classified as ‘Others’. Drug classes categorised as ‘Others’ are displayed with an asterisk in the supplements

**ATC-classification**		**Frequency**	
Analgesics	13.8%	Morphine	47
		Fentanyl	39
Psycholeptics	12.5%	Oxazepam	39
		Temazepam	31
Antidepressants	8.8%	Amitriptyline	15
		Nortriptyline	8
Antihistamines	7.5%	Clemastine	23
		Levocetirizine	4
Antiepileptics	6.3%	Pregabaline	24
		Clonazepam and gabapentin*	3
Antiemetics and antinauseants	5.0%	Granisetron	69
		Palonosetron	16
Diuretics	5.0%	Hydrochlorothiazide	41
		Furosemide	41
Antibacterials	3.8%	Ciprofloxacin	28
		Erythromycin and fusidic acid*	1
Antineoplastics	3.8%	Palbociclib	4
		Nilotinib and procarbazine*	1
Endocrine therapy	3.8%	Enzalutamide	15
		Ciclosporin and thalidomide*	1
Muscle relaxants	3.8%	Baclofen	4
		Suxamethonium	2
Urologicals	3.8%	Oxybutynin	5
		Fesoterodine and tolterodine*	1
Antiarrhythmics	2.5%	Amiodarone	2
		Disopyramide	1
Calcium channel blockers	2.5%	Diltiazem	10
		Verapamil	4
Corticosteroids	2.5%	Dexamethasone	62
		Prednisone	6
Drugs for obstructive airway diseases	2.5%	Ipratropium	19
		Tiotropium	18
Gastro-intestinal agents	2.5%	Metoclopramide	91
		Atropine	4
Others	10.0%	Pramipexol	10
		Clopidogrel	9

*ATC* anatomic therapeutic chemical; *DDD* defined daily dose

*Same frequency was observed for both drugs

Of the 457 different co-medications, 80 (17.8%) are DDI perpetrators of oxycodone according to the four consulted drug interaction databases. Of these 80 perpetrators, 65 (81.3%) were considered clinically relevant. Granisetron was the most prevalent clinically relevant perpetrator with a frequency of 23.6%. Table 3 presents the 15 most frequently observed clinically relevant perpetrators of oxycodone.

**Table 3:** Top 15 most observed clinically relevant perpetrator drugs (*n* = 254)

				**Associated ADR **					
	**Drug**	**Drug group based on ATC code**	**Frequency (%)**	**Influence in CYP3A4**	**Risk CNS depression**	**Risk respiratory depression**	**Risk serotonergic syndrome**	**Influence on perpetrator efficacy**	**Constipation and urinary retention**
1	Granisetron	Antiemetics and antinauseants	23.6				↑		
2	Dexamethasone	Corticosteroids	22.8	↑					
3	Hydrochlorothiazide	Diuretics	16.5					↓	
4	Furosemide	Diuretics	13.8					↓	
5	Oxazepam	Psycholeptics	13.0		↑	↑			
6	Ciprofloxacin	Antibacterials	10.6	↓					
7	Temazepam	Psycholeptics	10.2		↑	↑			
8	Clemastine	Antihistamines	7.9		↑				
9	Tiotropium	Drugs for obstructive airway diseases	7.1						↑
10	Ipratropium	Drugs for obstructive airway diseases	6.7						↑
11	Palonosetron	Antiemetics and antinauseants	6.3				↑		
12	Amitriptyline	Antidepressants	5.9		↑		↑		
13	Enzalutamide	Endocrine therapy	5.5	↑					
14	Lorazepam	Psycholeptics	5.1		↑	↑			
15	Pramipexole	Anti-parkinson drugs	3.9					↑	

*ADR* adverse drug reaction, *ATC* anatomic therapeutic chemical, *CNS* central nervous system, *CYP* cytochrome P450; ↑ = enhancement or induction; ↓ = reduction or inhibition

In 21 out of 254 oxycodone treatment episodes (8.3%), 23 double interactions were observed (Table 4). Of these, nine were double interactions with CYP inhibitors, and 14 were double interactions with CYP3A4 inducers. Six out of nine double interactions with inhibitors consisted of a combination of CYP2D6 and CYP3A4 inhibitors, two involved concomitant use of two CYP3A4 inhibitors, and one involved two CYP2D6 inhibitors. With regard to double interactions with inducers, 11 out of 14 concerned the combination of dexamethasone and prednisolone.

**Table 4:** Frequency of double interactions among oxycodone treatment episodes (*n* = 23)

**Double interactions with inhibitors**		
**CYP3A4 inhibitor**	**CYP2D6 inhibitor**	**Frequency**
Ciprofloxacin	Darifenacin	1
Ciprofloxacin	Mirabegron	2
Fluoxetine	Fluoxetine	1
Verapamil		
Netupitant	Paroxetine	1
Palbociclib	Venlafaxine	1
Ciprofloxacin	NA	1
Fluconazole		
Ciprofloxacin	NA	1
Nilotinib		
NA	Darifenacin	1
	Mirabegron	

**Double interactions with CYP3A4 inducers**		
**CYP3A4 inducers**		**Frequency**
Dexamethasone	Prednisolone	11
Enzalutamide	Prednisolone	1
Dexamethasone	Rifampicin	1
Dexamethasone	Enzalutamide	1

*NA* not applicable

## Discussion

This study is the first to describe the frequency of DDI perpetrators of oxycodone in patients with cancer. Additionally, the frequency of double interactions involving two CYP3A4 and/or CYP2D6 inducing or inhibiting drugs was assessed. In 89.4% of the oxycodone treatment episodes, at least one pharmacologically relevant perpetrator was found. Of these, 92.5% concerned a clinically relevant perpetrator. Of the 80 different perpetrators that were observed, 65 (81.3%) were considered both pharmacologically and clinically relevant. Granisetron and dexamethasone were the most frequently observed clinically relevant perpetrators (23.6% and 22.8%, respectively).

In our cohort, perpetrators from the ATC classes ‘analgesics’ and ‘psycholeptics’ were most frequently observed. Majority of clinically relevant perpetrators involved pharmacokinetic interactions. Some perpetrators were considered pharmacologically relevant, but not clinically relevant. The combination of oxycodone with other analgesics is intentional and generally closely monitored and is therefore not considered clinically relevant. However, nortriptyline and amitriptyline are considered clinically relevant perpetrators, since they are more commonly prescribed for depression in patients with cancer [20]. In addition, metoclopramide and domperidone are also not considered clinically relevant perpetrators, since the combination of oxycodone with these drugs is standard care for oxycodone-induced nausea, which affects up to 40% of this population [20]. A potential decrease in the effectiveness of metoclopramide and domperidone is managed by dosing according to clinical effect. Other antiemetics indicated for high-emetogenic chemotherapy, i.e. 5HT3-antagonists and neurokinin antagonists, are considered clinically relevant perpetrators, since these drugs are not used to treat oxycodone-induced nausea.

Both granisetron and dexamethasone are implemented in oncological treatment protocols, and their combination with oxycodone is frequently observed. However, concomitant use with oxycodone can result in severe ADRs. For instance, co-administration of oxycodone and granisetron can increase the risk of serotonin syndrome [21]. Moreover, dexamethasone can decrease oxycodone concentrations through CYP3A4 induction [22]. According to Hoeben et al., patients treated with 15 mg instant release oxycodone experienced a lower pain response compared to those treated with 10 mg instant release oxycodone [23]. Therefore, when a CYP3A4 inducer is used concomitantly with oxycodone, higher doses of oxycodone may be required. According to the prescribing information of oxycontin, patients should be closely monitored for life-threatening respiratory depression when the dosage is increased [15]. Hence, when dexamethasone is deprescribed, exposure to oxycodone increases and may result in adverse effects [21].

In total, 14 double interactions with CYP3A4 inducers were found in 254 oxycodone treatment episodes, of which 11 involved the combination of dexamethasone, prednisolone, and oxycodone. This can be attributed to the widespread use of these corticosteroids in several oncological treatment protocols. In addition, 6 double interactions with a CYP3A4 and CYP2D6 inhibitor were found. There is a lack of pharmacovigilance regarding the concomitant use of multiple drugs that induce or inhibit CYP enzymes. Therefore, it is hardly surprising that we observed several double interactions in this study. The limited literature available describes that double interactions can significantly affect the plasma levels of affected drugs. For example, studies have reported additive inhibition when two inhibitors are concomitantly used, such as the effect of paroxetine (CYP2D6 inhibitor) and itraconazole (CYP3A4 inhibitor) on oxycodone levels; ciprofloxacin (CYP1A2 inhibitor) and fluconazole (CYP2C19 inhibitor) on metamizole metabolites and erythromycin (CYP3A4 inhibitor) and fluvoxamine (CYP1A2 inhibitor) on fluvoxamine and ropivacaine levels [10, 12–14]. However, not only drugs can affect multiple routes of the CYP metabolism, but also pharmacogenomics play an important role. For instance, up to 10% of the Caucasian population has a non-functional CYP2D6 enzyme. This might result in an exposure to high oxycodone levels when CYP3A4 is inhibited, since that is the only metabolic CYP route left [24]. Double interactions with oxycodone and two concomitantly used CYP2D6 inhibitors were also assessed. No studies investigated the impact of concomitantly used CYP2D6 inhibitors on the pharmacokinetics of oxycodone. A study reported a double interaction between duloxetine and mirabegron—both CYP2D6 inhibitors—and desipramine, a weak CYP2D6 substrate. The study suggested the possibility of competitive inhibition at the CYP2D6 enzyme for desipramine [25]. We hypothesise that a similar effect may occur with oxycodone as a substrate, given its inferior metabolism via the CYP2D6 pathway [9]. However, since CYP2D6 is not the main metabolic route, it is expected not to be of clinical relevance [8]. Currently, there is no literature available that describes the impact of multiple concomitantly used CYP inducers. In contrast to inhibitors, which block the activity of existing enzymes, inducers stimulate the synthesis of new enzymes. This process takes several days to weeks to fully manifest, whereas inhibition occurs immediately. Therefore, the effects of inhibition are easier to observe [25–27]. Double interactions are not yet included in the interaction compendia. Further research is needed in order to determine whether they have to be included.

A limitation of this study is the potential for cognitive bias in the assessment of the clinical relevance of observed perpetrators by experts [28]. Ideally, the clinical relevance of perpetrators would be established and documented by interaction compendia. However, there is currently a lack of standardisation and classification of DDIs among compendia [29]. Another limitation is that not all patients consented to having their medical information shared with healthcare professionals through the National Exchange Point. In 2018, approximately 60% of patients in the Netherlands consented to their medical information being shared [30]. Furthermore, clinical outcomes were outside the scope of this study. Therefore, no data regarding side effects or toxicological cases are available. Strengths of this study include the reviewing and verifying the database by a secondary investigator, ensuring the reliability of data collection. Additionally, the cohort included a large number of patients—all oncology and haematology patients in the hospital—over a 1-year period. Pharmacy records from both the hospital and public sector were reviewed for each patient, providing a comprehensive overview of perpetrator frequency among patients with cancer. Furthermore, an experienced oncologist and a clinical pharmacologist were involved in this study to assess the clinical relevance of the observed perpetrators.

## Conclusions

A high frequency of pharmacologically and clinically relevant DDI perpetrators of oxycodone was observed in our cohort. In approximately nine out of ten times when patients with cancer were prescribed oxycodone for ≥ 5 consecutive days, patients received a pharmacologically relevant DDI perpetrator of oxycodone. Nearly all pharmacologically relevant DDI perpetrators were considered clinically relevant. Moreover, a high number of double interactions involving oxycodone was observed. DDI monitoring might require optimisation in order to improve medication safety of patients with cancer taking oxycodone.

### Supplementary Information

Below is the link to the electronic supplementary material.

Supplementary file1 (PDF 64 KB)

Supplementary file2 (PDF 31 KB)

Supplementary file3 (TIFF 1191 KB)

Supplementary file4 (TIFF 1191 KB)

Supplementary file5 (TIFF 1191 KB)

## Data Availability

The authors confirm that the data supporting the findings of this study are available within the article and its supplementary materials.
