# Supplementary material for: Drug-drug interaction perpetrators of oxycodone in patients with cancer: frequency and clinical relevance
Source: Eur J Clin Pharmacol. 2024 Jan 13;80(3):455–64. doi: 10.1007/s00228-023-03612-2 (PMC10873430; doi:10.1007/s00228-023-03612-2)
Supplement: Supplementary file 1 — Supplementary file1 (PDF 64 KB) [file 228_2023_3612_MOESM1_ESM.pdf]

Drug	ATC-category of the drug	Number of encounters in oxycodone treatment episodes	Frequency (%)	Relevant interaction?
**Metoclopramide**	Drugs for functional gastro-intestinal disorders	89	35.0	No
**Granisetron**	Antiemetics and antinauseants	60	23.6	Yes
**Dexamethasone**	Corticosteroids	58	22.8	Yes
**Morphine**	Analgesics	44	17.3	No
**Hydrochlorothiazide**	Diuretics	42	16.5	Yes
**Furosemide**	Diuretics	35	13.8	Yes
**Fentanyl**	Analgesics	34	13.4	No
**Oxazepam**	Psycholeptics	33	13.0	Yes
**Ciprofloxacin**	Antibacterials for systemic use	27	10.6	Yes
**Temazepam**	Psycholeptics	26	10.2	Yes
**Pregabalin**	Antiepileptics	23	9.1	Yes
**Clemastine**	Antihistamines for systemic use	20	7.9	Yes
**Tiotropium**	Drugs for obstructive airway diseases	18	7.1	Yes
**Ipratropium**	Drugs for obstructive airway diseases	17	6.7	Yes
**Piritramide**	Analgesics	17	6.7	No
**Palonosetron**	Antiemetics and antinauseants	16	6.3	Yes
**Amitriptyline**	Antidepressiva	15	5.9	Yes
**Enzalutamide**	Endocrine therapy	14	5.5	Yes
**Lorazepam**	Psycholeptics	13	5.1	Yes
**Codeine**	Analgesics	10	3.9	No
**Pramipexole**	Anti-parkinson drugs*	10	3.9	Yes
**Clopidogrel**	Anti trombotic agents*	9	3.5	Yes
**Diazepam**	Psycholeptics	9	3.5	Yes
**Spironolactone**	Diuretics	9	3.5	Yes
**Tramadol**	Analgesics	9	3.5	No
**Haloperidol**	Psycholeptics	8	3.1	Yes
**Netupitant**	No ATC classification*	8	3.1	Yes
**Nortriptyline**	Antidepressiva	8	3.1	Yes
**Citalopram**	Antidepressiva	7	2.8	Yes
**Levetiracetam**	Antiepileptics	7	2.8	Yes
**Zolpidem**	Psycholeptics	7	2.8	Yes
**Bumetanide**	Diuretics	6	2.4	Yes
**Fluconazole**	Antimycotics for systemic use	6	2.4	Yes
**Ondansetron**	Antiemetics and antinauseants	6	2.4	Yes
**Prednisone**	Corticosteroids	6	2.4	Yes
**Domperidone**	Drugs for functional gastro-intestinal disorders	5	2.0	No

**Palbociclib**	Antineoplastic agents	5	2.0	Yes
**Baclofen**	Muscle relaxants	4	1.6	Yes
**Buprenorphine**	Analgesics	4	1.6	No
**Diltiazem**	Calcium channel blockers	4	1.6	Yes
**Levocetirizine**	Antihistamines for systemic use	4	1.6	Yes
**Metamizole**	Analgesics	4	1.6	No
**Oxybutynin**	Urologicals	4	1.6	Yes
**Paroxetine**	Antidepressiva	4	1.6	Yes
**Sumatriptan**	Analgesics	4	1.6	No
**Venlafaxine**	Antidepressiva	4	1.6	Yes
**Verapamil**	Calcium channel blockers	4	1.6	Yes
**Clonazepam**	Antiepileptics	3	1.2	Yes
**Clonidine**	Psycholeptics	3	1.2	Yes
**Duloxetine**	Antidepressiva	3	1.2	Yes
**Gabapentin**	Antiepileptics	3	1.2	Yes
**Methadone**	Other nervous system drugs*	3	1.2	Yes
**Amiodarone**	Antiarrhythmics class I and III	2	0.8	Yes
**Atropine**	Drugs for functional gastro-intestinal disorders	2	0.8	Yes
**Cetirizine**	Antihistamines for systemic use	2	0.8	Yes
**Lormetazepam**	Psycholeptics	2	0.8	Yes
**Rifampicin**	Antimycobacterials	2	0.8	Yes
**Suxamethonium**	Muscle relaxants	2	0.8	Yes
**Almotriptan**	Analgesics	1	0.4	No
**Ciclosporin**	Endocrine therapy	1	0.4	Yes
**Disopyramide**	Antiarrhythmics class I and III	1	0.4	Yes
**Erythromycin**	Antibacterials for systemic use	1	0.4	Yes
**Fesoterodine**	Urologicals	1	0.4	Yes
**Fexofenadine**	Antihistamines for systemic use	1	0.4	Yes
**Indapamide**	Diuretics	1	0.4	Yes
**Meclozine**	Antihistamines for systemic use	1	0.4	Yes
**Mirtazapine**	Antidepressiva	1	0.4	Yes
**Naloxone**	All other therapeutic products*	1	0.4	Yes
**Nilotinib**	Antineoplastic agents	1	0.4	Yes
**Olanzapine**	Psycholeptics	1	0.4	Yes
**Oxcarbazepine**	Antiepileptics	1	0.4	Yes
**Pipamperone**	Psycholeptics	1	0.4	Yes
**Procarbazine**	Antineoplastic agents	1	0.4	Yes
**Promethazine**	Antihistamines for systemic use	1	0.4	Yes
**Quetiapine**	Psycholeptics	1	0.4	Yes

**Scopolamine**	Antiemetics and antinauseants	1	0.4	Yes
**Tapentadol**	Analgesics	1	0.4	No
**Tizanidine**	Muscle relaxants	1	0.4	Yes
**Tolterodine**	Urologicals	1	0.4	Yes
**Valproic acid**	Antiepileptics	1	0.4	Yes
