# Supplementary material for: Drug-drug interaction perpetrators of oxycodone in patients with cancer: frequency and clinical relevance
Source: Eur J Clin Pharmacol. 2024 Jan 13;80(3):455–64. doi: 10.1007/s00228-023-03612-2 (PMC10873430; doi:10.1007/s00228-023-03612-2)
Supplement: Supplementary file 2 — Supplementary file2 (PDF 31 KB) [file 228_2023_3612_MOESM2_ESM.pdf]

Drug	ATC-category of the drug	Number of single uses	Chronic uses (frequency; %)
Alfentanil	Anaesthetics	1	0 (0.0)
Atropine	Drugs for functional gastro-intestinal disorders	2	2 (0.8)
Ciprofloxacin	Antibacterials for systemic use	1	27 (10.6)
Clemastine	Antihistamines for systemic use	3	20 (7.9)
Dexamethasone	Corticosteroids	5	58 (22.8)
Diazepam	Psycholeptics	1	9 (3.5)
Domperidone	Drugs for functional gastro-intestinal disorders	1	5 (2.0)
Fentanyl	Analgesics	5	34 (13.4)
Furosemide	Diuretics	6	35 (13.8)
Granisetron	Antiemetics and antinauseants	10	60 (23.6)
Haloperidol	Psycholeptics	1	8 (3.1)
Hydrochlorothiazide	Diuretics	1	42 (16.5)
Ipratropium	Drugs for obstructive airway diseases	2	17 (6.7)
Lorazepam	Psycholeptics	1	13 (5.1)
Metoclopramide	Drugs for functional gastro-intestinal disorders	4	89 (35.0)
Morphine	Analgesics	3	44 (17.3)
Ondansetron	Antiemetics and antinauseants	2	6 (2.4)
Oxazepam	Psycholeptics	6	33 (13.0)
Oxybutynin	Urologicals	1	4 (1.6)
Palonosetron	Antiemetics and antinauseants	1	16 (6.3)
Piritramide	Analgesics	15	17 (6.7)
Pregabalin	Antiepileptics	1	23 (9.1)
Scopolamine	Antiemetics and antinauseants	1	1 (0.4)
Temazepam	Psycholeptics	6	26 (10.2)
