# Supplementary figures and images for: Drug-drug interaction perpetrators of oxycodone in patients with cancer: frequency and clinical relevance
Source: Eur J Clin Pharmacol. 2024 Jan 13;80(3):455–64. doi: 10.1007/s00228-023-03612-2 (PMC10873430; doi:10.1007/s00228-023-03612-2)

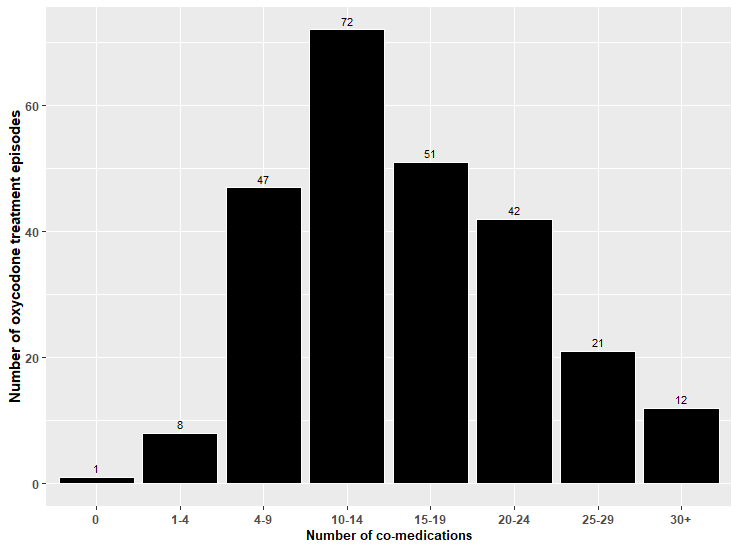

Supplement: Supplementary file 3 — Supplementary file3 (TIFF 1191 KB) [file 228_2023_3612_MOESM3_ESM.tiff]

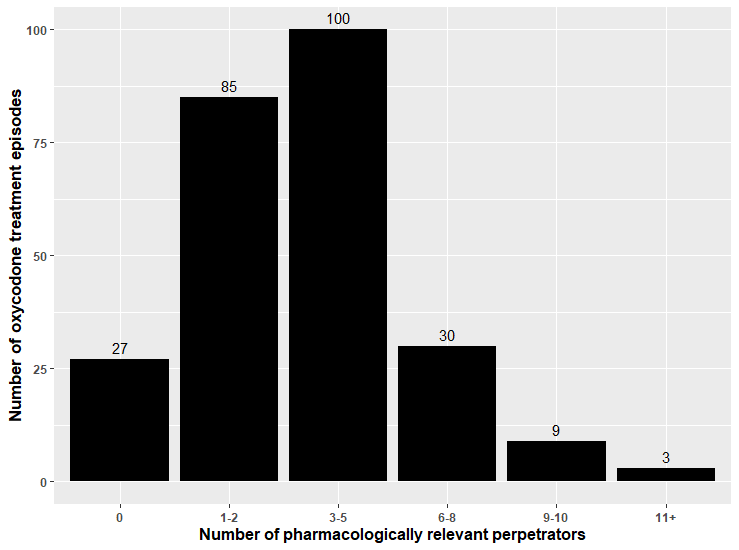

Supplement: Supplementary file 4 — Supplementary file4 (TIFF 1191 KB) [file 228_2023_3612_MOESM4_ESM.tiff]

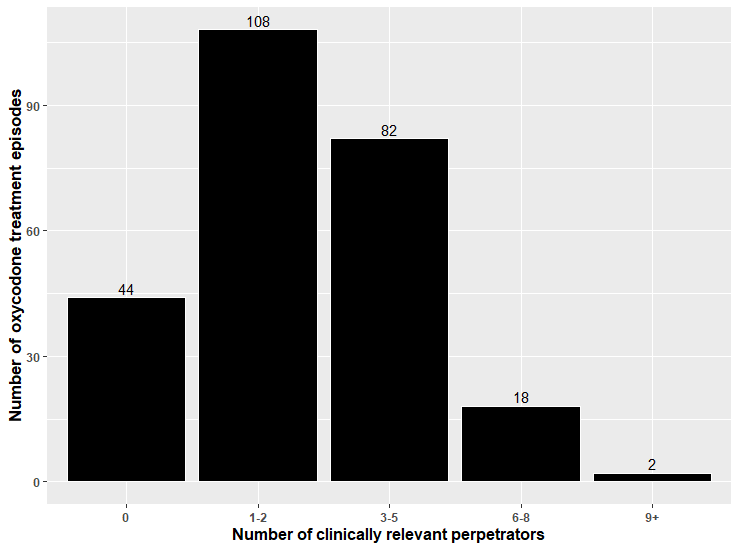

Supplement: Supplementary file 5 — Supplementary file5 (TIFF 1191 KB) [file 228_2023_3612_MOESM5_ESM.tiff]
